# Botulinum toxin as treatment for focal dystonia: a systematic review of the pharmaco-therapeutic and pharmaco-economic value

**DOI:** 10.1007/s00415-012-6510-x

**Published:** 2012-05-03

**Authors:** E. Zoons, M. G. W. Dijkgraaf, J. M. Dijk, I. N. van Schaik, M. A. Tijssen

**Affiliations:** 1 Department of Neurology, Academic Medical Centre, Amsterdam, The Netherlands; 2 Clinical Research Unit, Academic Medical Centre, Amsterdam, The Netherlands; 3 Movement Disorders, Department of Neurology AB 51, University Medical Centre Groningen (UMCG), PO Box 30.001, 9700 Groningen, The Netherlands

**Keywords:** Botulinum toxin, Focal dystonia, Effectiveness

## Abstract

Focal dystonia is a common, invalidating neurologic condition characterized by involuntary, sustained muscle contractions causing twisting movements and abnormal postures in one body part. Currently, botulinum toxin is the treatment of first choice. We performed a systematic review towards the pharmaco-therapeutic and pharmaco-economic value of botulinum toxin as treatment for focal dystonia, which yielded the following results. Botulinum toxin is the most effective treatment for reducing dystonic symptoms measured with dystonia-specific and general questionnaires, and pain in patients with focal dystonia. Seventy-one percent of patients with cervical dystonia had a reduction in neck pain compared to 12 % in placebo groups. Adverse events occur in 58 % of patients during treatment with botulinum toxin compared to 46 % treated with placebo. Especially dry mouth, neck weakness, dysphagia, and voice changes are common. Adverse events are usually mild and self-limiting. Health-related quality of life, measured with the SF-36 is 20–50 points lower in patients with focal dystonia compared to controls and the effect of botulinum toxin on health-related quality of life is unclear. Botulinum toxin treatment is expensive because the drug itself is expensive. Yearly costs for treating a patient with focal dystonia with botulinum toxin range from EUR 347 to EUR 3,633 and the gain in QALYs with BTX treatment is small. Focal dystonia impairs the productivity and the ability to work. At start of botulinum toxin treatment only 47–50 % was working. Botulinum toxin partly improves this. Overall, we conclude that botulinum toxin is an expensive drug with good effects. From a societal perspective, the costs may well weigh up to the regained quality of life. However, the available literature concerning costs, health-related quality of life and labor participation is very limited. An extensive cost-effectiveness study should be performed incorporating all these aspects.

**Electronic supplementary material:**

The online version of this article (doi:10.1007/s00415-012-6510-x) contains supplementary material, which is available to authorized users.

## Introduction

Dystonia is a syndrome characterized by involuntary, sustained muscle contractions causing twisting movements and abnormal postures [32]. Dystonia is the third most common movement disorder (annual incidence: 15–25 per 100,000) [11, 19] after Parkinson’s disease [36] and tremor [20]. Dystonia can be classified based on topographic distribution, including focal dystonia (one body region), segmental dystonia (two or more adjacent regions), multifocal dystonia (two or more nonadjacent regions), hemidystonia (ipsilateral arm and leg) and generalized dystonia [32]. Focal dystonia (FD) is the most common form of dystonia and can occur in every body part. Most often focal dystonia occurs in the neck (cervical dystonia, CD), but it can also occur in the eyelids (blepharospasm, BPS) or hands (task-specific or focal hand dystonia) [32]. The treatment of first choice is botulinum toxin (BTX), a neurotoxin that blocks the neuromuscular signal transmission [32]. In this systematic review, we assess the pharmaco-therapeutic value of botulinum toxin as treatment for FD including health-related quality of life. In addition, a systematic review of the pharmaco-economic value is performed.

## Methods

Relevant systematic reviews, randomized controlled trials (RCTs), and observational studies were identified by systematic searches in the Cochrane database, the Database of Abstracts of Reviews of Effects (DARE), Medline (from year of publication 1966 until November 2011), and Embase (from year of publication 1980 until November 2011). Language was limited to Dutch, English, German, and French. Articles concerning animal studies were excluded. Economical studies were sought in the economic evaluations of the Cochrane library, Medline, Embase and the NHS Economic Evaluation Database (NHS EED). Search terms that we used were a combination of “focal dystonia”, “cervical dystonia”, “blepharospasm”, “writer’s cramp”, “hand dystonia”, “oromandibular dystonia” or “Meige's syndrome” and “botulinum toxin”, “botulinum toxin A”, “botulinum toxin B”, “Dysport”, “Botox” or “Xeomin”. We searched for relevant systematic reviews first. Identified reviews were assessed for their methodological quality by two reviewers (JMD and INVS) using a standardized Dutch form that was developed by the Dutch Institute for Healthcare Improvement (CBO) and the Dutch Cochrane Centre [1]. In case the two reviewers disagreed on the quality of the articles, articles were discussed with the other authors until consensus was reached. Reviews that were assessed as good were used, and than additional case-cohort and observational studies were not included. If a review did not cover all the available literature, additional RCTs were sought from the end date of the literature search of the most recent review and assessed for their quality. In case there were no good reviews or RCTs, case-cohort or observational studies were sought and used. The available literature differs substantially for several relevant aspects of botulinum toxin treatment. Therefore, the best available literature was sought for each of these relevant topics. The topics discussed in this review are: favorable effects, adverse events, health-related quality of life, costs, and labor and/or production loss. Because of the available literature, the search results were divided into two parts: a therapeutic part (favorable effects and adverse events; most available literature) and an economical part (quality of life, costs and labor participation; less available literature). The results of the searches are depicted in two flow-charts, see flow-chart 1 and flow-chart 2. Main reasons to exclude articles or to not assess articles at full-text were: double search results, the article concerned another disorder, the article concerned another treatment, diagnostics or intervention or mentioning of the topics at interest (e.g., adverse events, costs or quality of life) only in the discussion part of the abstract. At the end of the article, we added an evidence paragraph stating the used articles and their evidence level per subheading.

To allow an adequate comparison of the costs reported in different cost-effectiveness studies, we adapted the costs to the Dutch situation according to the existing international guideline for good research practices for measuring drug costs [25]. Costs were adapted to Dutch euros in the year 2009. We used consumer price index values for the local currency to derive the cost equivalents for 2009 (Institut National de la Statistique et des études économiques, http://www.bdm.insee.fr; Statistisches Bundesamt Deutschland, http://www.destatis.de; Office for National Statistics, http://www.statistics.gov.uk; all accessed late December 2010). Cost estimates based on local currencies from before the introduction of the euro in 2001 were converted into euro. Subsequently, purchasing power parities for actual individual consumption (Organisation for Economic Cooperation and Development; stats.oecd.org) were used to convert the price-indexed estimates to ‘Dutch’ euros.

## What are the favorable effects of BTX treatment?

Favorable effects of BTX have been measured in several ways in different studies. Three frequently used outcome measures in studies were relief of dystonic symptoms, (self)-reported improvement of symptoms, and pain relief.

### Dystonic symptoms

Patients with FD improved significantly with BTX treatment when symptoms were measured with dystonia-specific scoring instruments [30, 33, 34, 37]. In the identified studies, the Tsui scale and Toronto Western Spasmodic Torticollis Rating scale (TWSTRS) were used for CD [30] and the blepharospasm rating scale [BRS; also referred to as the Jankovic Blepharospasm Rating scale (JBRS)], the Blepharospasm Disability scale (BDS) and the Blepharospasm Disability Index (BSDI) were used for BPS [34, 37]. Improvement was detected for changes in the mean scores on the Tsui and TWSTRS for CD, the JBRS, BDS, and BSDI for BPS and sound spectrograms for spasmodic dysphonia (dystonia of the vocal cords), but also for the proportion of patients with a clinically relevant difference on these scales (e.g., ≥3 points improvement on the Tsui scale or ≥20 % improvement on the TWSTRS). Improvement on the above named scales has been shown in studies using BTX type A and B in patients with different forms of FD [30, 33, 34, 37]. Several studies demonstrated that the effectiveness of BTX type A on the relief of dystonic symptoms in patients with CD showed no significant difference compared to type B [30]. The effect of BTX on dystonic symptoms lasted at least 12–16 weeks in both patients with CD and in patients with BPS consistent with the 12 week treatment interval used in clinical practice for both treatment groups [30, 34, 37]. Trihexyphenidyl, an anticholinergic drug, is the most commonly used drug, besides BTX, for the treatment of FD. This drug has been compared to BTX type A in patients with CD. The effect was measured using the Tsui and the TWSTRS. Trihexyphenidyl was significantly less effective compared to BTX in improving symptoms on these scales [3].

In a large review, no systematic reviews or RCTs evaluating other drugs for the treatment of focal dystonia, such as antipsychotics, dopamine (ant-)agonists or serotonin (ant-)agonists were identified [30].

### (Self-)reported improvement

Positive effects have also been shown on more general questionnaires measuring improvement of symptoms according to the patient or physician (e.g., Patient global assessment of change) in patients with CD and BPS in studies comparing BTX type A and B to placebo [3, 30]. Physicians reported a beneficial effect on symptoms of dystonia in 37–65 % of patients with CD (depending on dose) and 94–97 % of patients with BPS treated with BTX compared to 17–30 % in the various placebo groups [30, 34]. Patients with CD considered BTX treatment beneficial in 43–68 % (depending on dose) compared to 10–22 % of the placebo group [30]. In BPS, 87–97 % of patients in the BTX group reported a beneficial effect, compared to 25 % of patients in the placebo group [34]. In the only methodologically good RCT in patients with writer’s cramp that was identified in a large review [30] 70 % of patients in the BTX group expressed the wish to continue treatment compared to 32 % of patients in the placebo group [18]. Different dosages of both BTX type A and B have been compared to placebo in patients with CD. The proportion of patients that reported improvement was larger when higher dosages were used. Physicians also reported improvement in a larger proportion of patients when higher dosages of BTX were used. This came at the cost of more adverse events (see “Adverse events”). Unfortunately, no direct comparisons have been made between the low- and high-dose groups [30, 34].

### Pain

Pain is an important problem in patients with CD but not in patients with other forms of FD [32]. Seventy-five percent of CD patients suffer from invalidating neck pain [32]. Treatment with both BTX type A and B significantly reduced neck pain: 71 % of patients treated with BTX had a reduction in neck pain versus 12 % of patients treated with placebo [30]. BTX was more effective than trihexyphenidyl in reducing neck pain: 2–3 points improvement on TWSTRS-pain in the BTX group versus 1 point in the placebo group [3].

## What are the adverse events of BTX treatment?

Adverse events are common in BTX trials in patients with FD. In the identified articles, they were only reported in patients with CD and WC, but they may occur in all forms of FD. In a large review, 58 % of patients with CD treated with BTX type A experienced adverse events compared to 46 % treated with placebo [30]. The most common adverse event was dry mouth in 19–41 % of patients treated with BTX type A and 22–80 % of patients treated with BTX type B [30]. Other common adverse events were neck weakness (18 %), dysphagia (3.4–19.4 %) and voice change or hoarseness (7 %) [8, 30]. Incidence of adverse events increased with increasing dosages, both for BTX type A and B. Most adverse events were mild and self-limiting [30]. One of the most feared possible adverse events of BTX treatment is systemic weakness. According to a recent literature review, this mainly occurred in patients treated with injections exceeding 600 units of Botox (e.g., spastic paraparesis). Only one case of systemic weakness in a patient with CD has been reported in the literature. This female patient received 650 U of Dysport and developed fatigue, limb weakness, and dysphagia after 1 week. The complaints lasted for 6 months [9]. Death, although warned for by the FDA, is an extremely rare side-effect and was not mentioned in any of the identified studies, nor did we identify any case reports of fatalities in patients with dystonia.

In CD patients treated with trihexyphenidyl, adverse events are more common compared to BTX treated CD patients [3].

In 40 patients with WC weakness in the hand was more common in the BTX group (18/20; 90 %) than in the placebo group (2/19; 11 %), although there was no *p* value reported. Pain at the injection site also was a common adverse event in these patients, but rates were comparable between the BTX group (1/20; 5 %) and placebo group (3/19; 16 %) [18].

Another unfavorable event associated with BTX treatment is the possibility of antibody formation due to the injection of a foreign protein. In a large series, 17 of 303 patients became secondary non-responders. Nine of those 17 (3 % of total) had detectable antibodies [29]. Primary failure to BTX because of resistance is very rare [29].

## How is the quality of life of patients with focal dystonia?

Quality of life is measured with and quantified by general (e.g., SF-36 or EQ5D) or disease-specific quality of life questionnaires and is therefore different from self-reported well-being. Scores on quality of life questionnaires can be used to compare the disease burden of different conditions. Furthermore, gain in quality of life can be weighed against treatment costs [14].

Health-related quality of life of patients with FD was significantly worse compared to the general population in all identified studies [2, 6, 12, 15, 16, 23, 24, 26, 28, 31, 35]. In most studies, health-related quality of life was measured with the SF-36, a questionnaire consisting of 36 items that can be divided into eight domains. Both total and domain scores can range from 0 to 100 [38]. Patients with FD scored 20–50 points lower on the total score compared to the general population [2, 6, 12, 16, 23, 26, 28]. Scores were comparable to patients with multiple sclerosis, Parkinson’s disease or stroke [6, 24]. Scores were lower on all SF-36 domains [12, 16, 23, 26, 28]. Patients with CD scored worse on the bodily pain domain compared to patients with other forms of FD [16, 23, 24]. A study in patients with spasmodic dysphonia found no differences in SF-36 scores between patients and normative scores for the general population, but, using a more disease-specific voice-related quality of life questionnaire (V-RQOL), quality of life was markedly reduced in patients [31].

The most important determinants of worse health-related quality of life were depression and anxiety [2, 15, 24, 26, 28]. In one study, 47 % of patients with BPS and CD fulfilled the criteria for depression [23]. Predictors for lower health-related quality of life were more severe dystonia [2, 23, 26], longer disease duration [2], pain [23, 24], higher age [26], female gender [6, 23], low self-deprecation [2], being single [2] and being unemployed [12, 24]. On the other hand, longer disease duration [23, 31] and higher age [12] were also associated with better health-related quality of life.

The effect of BTX on health-related quality of life is unclear and studies differ on this point. This is partly due to short follow-up: 4 weeks follow-up in two studies [23, 28], 12 weeks in one study [16], and 6 months in one study [12]. For BTX treatment, this is still relatively short, since one treatment cycle is 3 months. One study in patients with CD showed a significant improvement on all SF-36 domains after BTX treatment, for some domains even to the level of the general population [28]. In another study there was only a mild improvement in SF-36 domain scores in patients with CD. The health-related quality of life of patients with BPS did not improve with BTX treatment [23]. Two studies showed a mild improvement in SF-36 scores in patients with all types of dystonia when BTX was working (after ~4 weeks) and a decline when the effect wore off (after ~12 weeks) [12, 16]. These changes were statistically significant in only one of those studies in patients with CD and BLS [16].

One study reported score distribution of the different EQ5D dimensions in patients with focal dystonia, compared both to patients with non-focal dystonia and the general population. They included 93 patients with different forms of focal dystonia, of which 81 patients were being treated with BTX. Overall, patients with focal dystonia scored significantly worse on all five dimensions compared to the general population. The overall utility index improved after BTX treatment and worsened again as treatment effects wore off [12].

## What are the costs of BTX treatment?

Yearly costs of botulinum toxin treatment for FD ranged from EUR 347 to EUR 3,633, depending on number of treatment sessions per year and type of dystonia treated [4, 5, 7, 10, 13]. The lowest costs were for treating BPS patients three times a year and the highest for treating CD patients five times a year [5, 10]. Yearly costs for not treating FD patients were estimated to be between EUR 248 and EUR 1,014 and consisted of visits to the outpatient clinic, other medication and physiotherapy, among others [4, 13]. Most studies only incorporated direct medical costs that mainly consist of the costs of the toxin itself. A recent study looked more extensively at the costs of treating patients with neurological conditions, including focal dystonia, with BTX. The authors included costs of the toxin (EUR 154.36 for 100 IU Botox and EUR 215 for 500 IU Dysport), salaries of the treating physician, assisting nurse and secretary, needles, EMG equipment and social costs (transportation by taxi with an accompanying person). They estimated that the daily costs were EUR 1.30 ± 0.30 for treating a BLS patient, EUR 1.52 ± 0.73 for a patient with oromandibular dystonia, EUR 3.28 ± 0.86 for CD and EUR 1.15 ± 0.35 for occupational dystonia (not further specified). This leads to yearly costs ranging from EUR 419.75 for occupational dystonia to EUR 1,197.20 for cervical dystonia. Subjective improvement in these patients varied between 2.2 and 2.4 on a four-point rating scale [5].

In cost-effectiveness studies, benefits are usually measured in gained quality adjusted life years (QALYs). The range of yearly gain in QALYs following BTX treatment was 0.015–0.114, depending on the type of valuation (time trade-off or visual analogue scale) and type of focal dystonia [7, 13]. Saved costs for remaining at work (direct non-medical costs) and not buying over the counter drugs (indirect non-medical costs) were not assessed. Extra costs per gained QALY ranged from EUR 5,301 to EUR 94,770, depending on the baseline health-related quality of life, the valuation methods of health status applied (visual analogue scoring versus time trade-off based elicitation of preferences), treatment costs and type of FD, suggesting that health care efficiency may substantially differ between patient groups. This could indicate that limits for treatment indications must be set in the future [7, 13]. Based on the type of patients treated, average BTX dose, dosing interval and average gain in QALYs, an estimate of EUR 40,000 per gained QALY is probably most realistic for the Dutch situation.

All studies mentioned that the costs of BTX treatment for FD mainly consist of the costs of the toxin itself, since BTX is an expensive drug [4, 5, 7, 10, 13, 40]. Other cost drivers include for example material, personnel costs, and travel expenses [5]. One study evaluated whether the costs could be lowered by getting an outreach nurse practitioner to administer the BTX at the patient’s home. This was at least as effective and safe as treatment at the outpatient clinic, and was valued significantly higher by patients. The costs of the visit (excl the BTX) were halved, but the difference on total costs was not significant, since BTX was the main cost driver [40].

## How do focal dystonia and BTX treatment influence labor participation?

Indirect costs that originate when patients are not able to work have not been evaluated in any of the above-named studies. Therefore, we looked for articles discussing the effect of FD and BTX treatment on work. We identified three Scandinavian studies in patients with CD.

Before the first symptoms of dystonia became apparent, 78.7–84 % of patients were working [22, 27]. This percentage had dropped to 47–50 % at the start of BTX treatment and was 52 % after long-term BTX treatment [21, 22, 27]. Of the patients at work at the start of BTX treatment, 72 % remained employed. Of the patients on sick leave at the start, 67 % returned to work [27]. Fifty-three percent of patients reported a negative effect of CD on work and 68.9 % reported a negative effect on productivity [22]. In total, 18.9–47 % had to quit working or retire early because of dystonia [21, 22]. This effect was already noticeable at the age of 40 years. At the age of 50 years, only 25 % of patients were working compared to 62 % of the general population [21].

In CD patients, BTX had a better effect on work and productivity compared to oral medication. Also, the adverse effects of BTX had a smaller effect on work and productivity [22]. Important predictors of staying at work were younger age (<55 years) and longer education [27]. A predictor of being unemployed was neck pain [22].

## Discussion

The favorable effects of BTX treatment for patients with FD have been well described and are well known, as are the possible adverse events. BTX is the first treatment of choice when treating patients with different forms of FD. This is partly due to the favorable effects of BTX, but also because it is the best-studied treatment option in these patients. Over the last years, deep brain stimulation (DBS) has been an upcoming treatment for different forms of dystonia. We chose not to incorporate studies investigating DBS as a possible treatment for FD, since thus far DBS is used in a selected patient group where BTX treatment has failed. Furthermore, DBS as treatment for dystonia could be and has been a review subject itself [17, 39, 41].

The other topics we discuss (quality of life, costs, and labor participation) have been less well studied in patients with focal dystonia who are treated with BTX. To our knowledge, this is the first review gathering the available literature on these topics. The impact of FD on health-related quality of life appears to be large, but has, to our knowledge, never been reviewed before. Gathering the available evidence improves comparison of the disease burden of patients with FD to patients with other conditions. Studies evaluating the effect of focal dystonia on labor have only been performed in Scandinavian countries. These countries differ in their pension system and healthcare system from, for example, the Netherlands making it difficult to compare the data to the Dutch situation. The costs of BTX treatment for focal dystonia have not been reviewed before. Unfortunately, all studies include different cost components (see Table 1). Thus far, there has not been a cost-effectiveness study that incorporated all medical and non-medical costs. In the most complete and most recent cost study, QALYs were not evaluated, making it difficult to assess cost-effectiveness [5]. Based on data from the other studies, we estimated costs/QALY to be approximately EUR 40,000 for treating an FD patient with BTX, making BTX an expensive treatment. Indirect costs of lost production due to work absenteeism could not be retrieved from the reported studies, however, data on labor participation show that at least one-third of the patients stop working when the disease progresses, while being at work does not guarantee full productivity. Compared to oral medication, however, BTX seemed to exert a favorable influence on labor participation. From a societal perspective, however, the costs may well weigh up to the regained quality of life, making the drug affordable.

**Table 1:** Cost components in different cost studies

	Brefel-Courbon	Burbaud	Chadda	Dodel	Gudex	Whitaker
BTX	×	×	×	×	×	×
Clinic-related costs (e.g., outpatient visits)	×		×	×	×	×
Transportation and/or traveling costs		×			×	×
Needles and/or EMG equipment		×		×		
Salaries of medical personnel		×				×
Other medication	×				×	
Salaries of non-medical personnel		×				
Physiotherapy	×					
Diagnostic costs	×					
Patient lost time from work						×
Escort lost time from work						×
Administration			×			

The study has some limitations. First, as stated above, the information on health-related quality of life, treatment costs and labor participation in patients with focal dystonia is limited. This makes it difficult to draw any hard conclusions from the available literature. Second, most studies we included concern patients with cervical dystonia, the most common, and thus the most studied form of focal dystonia [32]. Even favorable effects and adverse events have not been well studied in other forms of focal dystonia. Third and last, it is always possible that we missed relevant references. We performed an extensive literature search, so we think it is unlikely we missed important references during our search. However, when available, we included reviews of sufficient quality and did not search for missing RCTs in the period covered by the review. We can not exclude that the reviews we included missed relevant references.

Concluding, BTX is the first treatment of choice for focal dystonia. BTX has large favorable effects and little adverse events. Available literature on the topics of health-related quality of life, costs, and labor participation is limited, but it appears that BTX is an expensive treatment that can improve quality of life and labor participation. A cost-effectiveness study that incorporates all direct and indirect medical and non-medical costs should be performed. In our opinion, the next step would be an international study evaluating all possible costs of BTX treatment including labor participation and quality of life. This study should include patients with different forms of focal dystonia. Since BTX is considered the first treatment of choice in most forms of focal dystonia, it would be unethical to perform a placebo-controlled trial, but new patients could be compared before and after BTX treatment.

## Evidence

Below is a summary of the evidence class of the articles we based this article on. Evidence is summarized per subheading. The articles represent the best available evidence.

### Favorable effects

One review covering literature up to February 2011 (class I) [30] and one additional RCT (class I) [37]. In the review, some subjects were covered by only one RCT, in that case the original study was used. The three studies concerned were all RCTs (class I) [3, 18, 33]. We identified one other RCT that was not mentioned in the review and that was of sufficient quality according to our method, so we incorporated this RCT as well [34].

### Adverse events

Four reviews (class I) [8, 9, 29, 30] covering the available literature up to February 2011 and one RCT (class I) [3] extracted from one of the reviews (see above).

### Quality of life

Four studies with a before-and-after design (class III) [12, 16, 23, 28] and seven cross-sectional studies (class IV) [2, 6, 15, 24, 26, 31, 35].

### Costs

Four cohort studies (class III) [4, 5, 10, 40] and two cross-sectional studies (class IV) [7, 13].

### Labor

Three cross-sectional studies (class IV) [21, 22, 27].

## Electronic supplementary material

Below is the link to the electronic supplementary material.

Supplementary material 1 (DOC 33 kb)
