# Supplementary material for: Botulinum toxin as treatment for focal dystonia: a systematic review of the pharmaco-therapeutic and pharmaco-economic value
Source: J Neurol. 2012 May 3;259(12):2519–26. doi: 10.1007/s00415-012-6510-x (PMC3506193; doi:10.1007/s00415-012-6510-x)
Supplement: Supplementary file 1 — Supplementary material 1 (DOC 33 kb) [file 415_2012_6510_MOESM1_ESM.doc]

**Flow-chart 1: therapeutic search (favorable effects and adverse events)**

Abstracts of RCT’s (February 2011 to November 2011) identified in Medline and Embase: 20

Assessed at full-text: 3 RCT’s

Included in review: 1

Not included: 2

DBS study: 1

No RCT: 1

Abstracts of reviews identified in Cochrane Database, DARE, Medline and Embase: 59

Assessed at full-text: 21 reviews

Included in review: 5

Not included: 16

Overlap of included studies: 10

Insufficient quality: 6

**Flow-chart 2: economical search (quality of life, costs and labour participation)**

Assessed at full text: 23 articles

Included in review: 19

Not included: 4

New QoL questionnaire: 1

DBS study: 1

No BTX treatment: 2

Abstracts of reviews identified in Cochrane Database, DARE, Medline and Embase: 0

Abstracts of RCT’s, case-control studies and observational studies identified in Medline and Embase: 459

Abstracts of articles identified in NHS EED: 2

Included in review: 1

Not included: 1

Double search result: 1
